# Effect of Physical Parameters and Training Load on Patellar Tendon Stiffness in Professional Athletes

**DOI:** 10.3390/diagnostics13152541

**Published:** 2023-07-31

**Authors:** Claudia Römer, Enrico Zessin, Julia Czupajllo, Thomas Fischer, Bernd Wolfarth, Markus Herbert Lerchbaumer

**Affiliations:** 1 Department of Sports Medicine, Charité Universitätsmedizin Berlin, 10115 Berlin, Germany; 2 Department of Radiology, Charité Universitätsmedizin Berlin, 10117 Berlin, Germany

**Keywords:** elastography, shear wave elastography, SWE, tendon, muscle, ultrasound

## Abstract

Background: Injuries of the patellar tendon commonly occur as a result of mechanical loading of the tendon during physical activity. Shear wave elastography (SWE) is an established technique for assessing tendon stiffness, and has good interindividual reliability. The aim of this study was to investigate the impacts of physical parameters and different sports on patellar tendon stiffness in professional athletes using SWE. Methods: Standardized patellar tendon SWE was performed in a relaxed supine position with a small roll under the knee (20° flexion) in 60 healthy professional athletes (30 female, 30 male). Multiple linear regression was performed for patellar tendon stiffness including gender, age, body mass index (BMI), and type of sport. Results: Patellar tendon stiffness showed no significant difference between female (3.320 m/s) and male (3.416 m/s) professional athletes. Mean age (female: 20.53 years; male: 19.80 years) and BMI (female: 23.24 kg/m^2^; male: 23.52 kg/m^2^) were comparable. Female professional athletes with oral contraceptive (OC) intake showed higher patellar tendon stiffness than athletes without OC intake (3.723 versus 3.017; *p* = 0.053), but not significantly. Conclusion: In professional athletes, there are no significant differences in patellar tendon stiffness according to gender, age, BMI and type of sport (handball, volleyball, soccer, sprint, hammer throw). Oral contraceptives may not have an impact on patellar tendon stiffness in female athletes. Further studies are necessary.

## 1. Introduction

Injuries of the patellar tendon (PT) commonly occur as a result of high mechanical loading of the tendon during physical activity. Athletes practicing sports involving jumping like volleyball or basketball have a lifetime risk of developing patellar tendinopathy of 50–55% [1,2,3]. In the general population, women, obese individuals and those with muscle weakness are known to have a higher risk of tendon injuries [4]. Possible underlying mechanisms include structural changes in elasticity and tendon thickness.

### 1.1. Tendon Physiology and Pathophysiology of Patellar Tendon

Tendons combine elastic and viscous characteristics when undergoing deformation in response to stress—known as viscoelasticity. Changes in this molecular structure have an impact on tendon stiffness and function [5]. If not given adequate time to recover, an overuse injury may result. Overuse leads to an imbalance between repetitive dysfunctional and micro-traumatic processes and basal repair mechanisms in tendon tissue [6]. Consequently, the molecular tendon structure (collagen crosslinking, extracellular matrix and vascular elements) weakens [6,7], which can be detected by SWE. If acute patellar tendinopathy is not treated adequately or persists, affected individuals may develop chronic tendinopathy. It has been shown that fibrin may organize and form adhesions between the tendon and paratenon, resulting in the thickening of the paratenon. Tenderness mechanic stress, like regular exercising, has been found to have a beneficial effect on age-related alterations and to counteract declines in muscle–tendon unit function [8].

Furthermore, musculoskeletal tissue expresses sex hormone receptors [9,10,11], which need to be considered in research more often, not only in professional athletes. Patellar tendon stiffness has been described to be lower in individuals with higher estrogen levels [12], and ACL stiffness has been altered dependent on estrogen level during menstrual cycle [13]; however, the observed study populations were small.

Lower tendon stiffness may lead to higher tension and stronger deformation due to high force impact. It has also been discussed that mechanical tendon changes may affect muscle response latency, which may be a relevant risk factor for musculoskeletal injuries [14,15]. The impact of jumping on the PT is 8 kN, and can be compared with forces of six to eight times body weight [16]. Athletes with recurring jumping movements (i.e., basketball and volleyball) are predisposed to PT injuries, especially chronic tendinopathy. Ultrasound B-Mode findings such as tendon thickening and structure changes (hypoechogenic changes) were described with low correlation to the appearance of PT pain syndrome [2,17]. Cook et al. described a higher prevalence of hypoechogenic areas in PTs of athletes in comparison to non-athletes [18]. Hypoechogenic areas occurred more often in male athletes and basketball athletes; however, these athletes were all free of symptoms [18], while stiffness of the patellar tendon was not measured. At the same time, ultrasound can detect tendon abnormalities before symptoms arising from patellar tendon pain syndrome [17,19]. The combination of B-mode ultrasound and power Doppler sonography does improve the clinical examination, and should be applied in elite athletes [3]. However, suspicious structural tendon areas can also be found in asymptomatic athletes, which can be an early sign of tendinopathy [3,20]. Morbus Osgood Schlatter or initial tendinopathy might be diagnosed by incident [20]. 

### 1.2. Diagnosis of Tendons’ Pathology Using Shear Wave Elastography

Shear wave elastography (SWE) is a technology used to quantify tendon stiffness, and shows potential in visualizing tendon changes, resulting in an improved diagnosis of tendinopathy [21], which leads to a higher precision of diagnosing and monitor tendinopathy in professional athletes nowadays. 

Professional athletes in jumping sports are at high risk for patellar tendinopathy and patellofemoral pain syndrome (PFPS) [22]. Patellar tendon thickness and cross-sectional area are correlated with PFPS [23]. Besides high training volume, lateral imbalances, resulting from early sport specialization, are leading causes for injuries in adolescent athletes and young adults [24,25]. At the same time, athletes with preceding knee injuries show a higher risk for re-injury [26]. Prospective examinations of untreated strength imbalances led to a higher incidence of muscle injury compared to athletes without lateral imbalances [27]. There are few data on patellar tendon stiffness changes assessed by shear wave elastography [21,28,29]. In a non-athlete population, patellar tendon stiffness was lower in subjects with moderate activity in comparison to sedentary subjects [28]. In patellar tendon tendinopathy, shear wave elastography values were significantly lower than in healthy tendons; however, the observed cohort were not professional athletes [21]. It is necessary to consider the causes of tendinopathy, whether there is an inflammatory process, as observable for example in diabetes patients, or whether there is a chronic overuse, as often seen in high training overload [30]. Patellar tendon stiffness in the dominant leg of professional soccer athletes has been found to be lower than in healthy individuals [29]. A long-term observation of soccer athletes has not been described, though decreased patellar tendon stiffness might be a sign of onset tendinopathy [21]. SWE is an established technique used to assess tendon stiffness with good interindividual reliability [31], and could be an important diagnostic method for use in prevention and rehabilitation. However, data for professional athletes and sport-specific analyses are missing. Therefore, we conducted a study aimed at investigating the potential of SWE to determine the impacts of physical parameters and different sports on patellar tendon stiffness in professional athletes. 

## 2. Methods

### 2.1. Study Population

The prospective study included 60 healthy professional athletes. The inclusion criteria were: (I) healthy professional athletes, (II) without any acute (<6 months) musculoskeletal, rheumatic or vascular comorbidities and no previous injuries of the patellar tendon, especially no PFPS, and (III) written informed consent to participate in the study. The following exclusion criteria were applied: tendon changes such as neovascularization, hypoechogenic areas and tendon thickening. The study was conducted in accordance with the Declaration of Helsinki and with the approval of the local ethics committee of Charité—University Medicine (ethical vote number EA2/162/19).

Baseline characteristics of study participants were acquired by a standardized questionnaire prior to the examination. There was a 30 min rest time due to the questionnaire survey before SWE measurement. On the day of the US-SWE examination, no training was performed. All examinations were jointly performed by a trained sonographer and a highly experienced radiologist who were blinded to the type of sport.

### 2.2. SWE Examination Protocol

**Figure 1:**
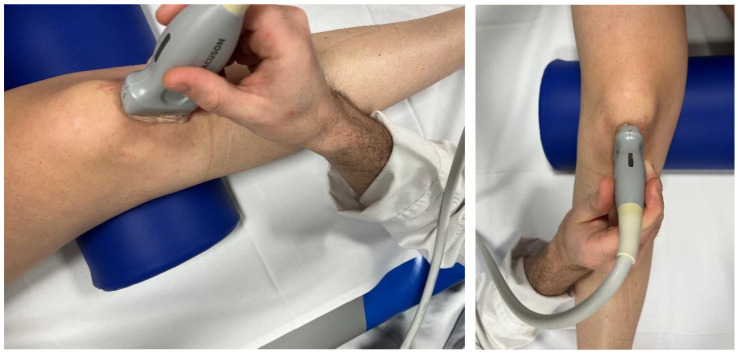
SWE examination of the right patellar tendon of a female professional athlete in relaxed supine position with a small roll under the knee (20° flexion).

US-SWE examinations were performed using a standardized protocol. For assessment of the PT, patients were positioned in a relaxed supine position with a small roll under the knee (resulting in a 20° flexion) (Figure 1). Prior to US-SWE, gray-scale B-mode US was performed in transverse and longitudinal planes to identify the optimal probe position for adequate tendon assessment. PT stiffness was captured at the central part of the patellar tendon. All examinations were performed using a high-end US system with a 4–10 MHz multifrequency linear array transducer and a center frequency of 7 Mhz (Acuson Sequoia, Siemens Healthineers, Erlangen, Germany). The US-SWE software (Virtual Touch™, Siemens Healthineers, Erlangen, Germany) allows for real-time measurement by using the Acoustic Radiation Force Impulse (ARFI) imaging technology for the quantification of shear wave speed.

**Figure 2:**
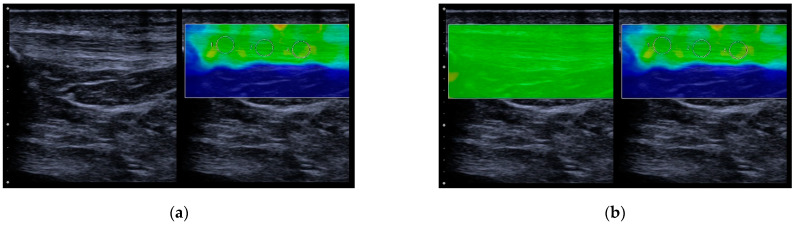
SWE images of the patellar tendon of a female professional athlete in B-mode and color-coded SWE mapping (**a**) and quality map of SWE measurement (**b**). The green quality map shows sufficient image quality (compared to red color map, which means inconclusive measurements (not shown)).

US-SWE examinations were performed in longitudinal orientation to depict the tendon and the area of interest in a single image (Figure 2). Using the 2D SWE approach, the examiner acquired four US images of the PT of the right leg of each professional athlete with a total of 600 consecutive SWE measurements in a 3 mm circular region of interest (ROI) placed in the center of each target tendon, avoiding areas of visible artifacts. Representative tendon stiffness is given as the median of 10 measurements and the corresponding interquartile range (IQR). Before ROI placement, shear wave velocity (SWV) as a surrogate for tissue stiffness was illustrated by color-coded SWE mapping. The standardized penetration depth was adapted to each participant for optimal visualization of the tendon and correct SWE measurement. Gain was not changed to avoid potential effects on US-SWE measurement.

### 2.3. Statistical Analysis

Multiple linear regression analysis of PT stiffness was performed using anthropometric parameters such as gender, age, BMI, and type of sport as input parameters. Continuous variables were tested for normal distribution using the Kolmogorov–Smirnov test. Not normally distributed variables are reported as median and interquartile range (IQR). A two-sided significance level of α = 0.05 was defined as appropriate to indicate statistical significance. All statistical analyses were performed using the SPSS software (IBM Corp., released 2019. IBM SPSS Statistics for Windows, Version 26.0. Armonk, NY, USA: IBM Corp.).

## 3. Results

### 3.1. Athletes’ Characteristics

A total of 60 professional athletes (30 female, 30 male) with a median age of 19.00 years (18.00–23.25) years were examined. The median body mass index (BMI) was 23.02 kg/m^2^ (21.90–24.43). No chronic diseases or acute health restrictions in general or of the lower limb joints were known. In particular, no patellar tendon injuries, symptoms of Osgood–Schlatter disease, or PFPS were reported. Nine female athletes took oral contraceptives. All professional athletes trained more than ten hours per week. Different types of sports were included (handball, *n* = 11; volleyball, *n* = 11; soccer, *n* = 21; hammer throw, *n* = 9; sprint, *n* = 8). Seven athletes in the oral contraceptive group (OC) took a combined oral contraceptive (with 0.02 to 0.03 mg ethinylestradiol), while two took a desogestrel preparation (0.075 mg). All athletes in the OC group took oral contraceptives for one year minimum. All athletes in OC and NOC confirmed that they have had a regular menstrual cycle of 28 ± 2 days for at least one year. The first day of the last menses was recorded by questionnaire. The day of examination was then allocated to a day in the menstrual cycle with days 1–7 being allocated to the early follicular phase (menses), days 8–14 being allocated to the late follicular phase and days 15–28 being allocated to the luteal phase, as the luteal phase is known to generally last fourteen days. 

### 3.2. Results of US-SWE in Professional Athletes

In the total study population of 60 professional athletes, mean patellar tendon stiffness was 3.165 m/s. No significant difference was found between female and male professional athletes (*p* > 0.05). Patellar tendon stiffness was highest in hammer throw athletes (3.61 m/s), followed by sprinters (3.50 m/s), soccer players (3.34 m/s), handball players (3.21 m/s), and volleyball players (3.04 m/s). Analysis of variance (ANOVA) for PT stiffness revealed no significant impacts of gender, age, BMI or type of sport. The results are compiled in Table 1 and Table 2. 

**Figure 3:**
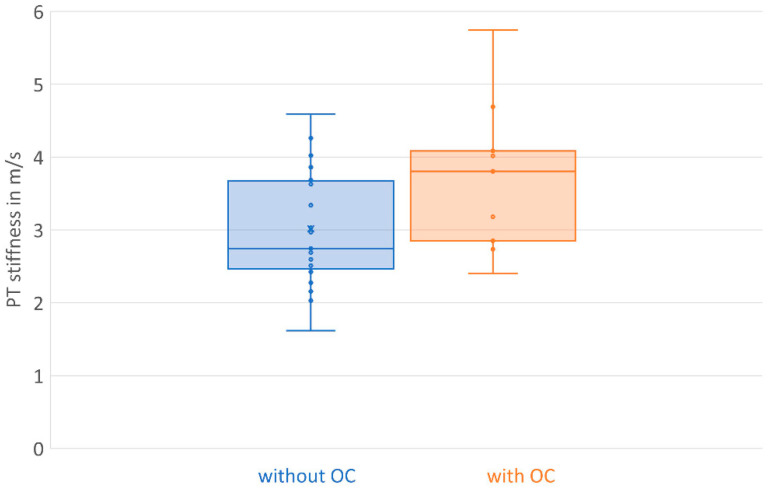
Boxplot of PT stiffness in female athletes without (blue) and with (orange) oral contraceptive intake.

Nine professional female athletes took an oral contraceptive pill. The subgroup analysis revealed higher patellar tendon SWE values in female athletes with oral contraceptive intake, but this was not significant (*p* = 0.053). The results are presented in Table 3 and Figure 3. Age and BMI were comparable in female athletes with and without oral contraceptive intake.

## 4. Discussion

SWE is a real-time ultrasound-based diagnostic technique for the noninvasive evaluation of soft tissue elasticity and stiffness by measurement of SWV. SWE has shown its potential and is already established for clinical use in breast, liver, thyroid and prostate imaging [32]. Dirrichs et al. found that shear wave elastography had higher sensitivity than B-mode and power Doppler US—the established clinical ultrasound modalities for the diagnostic examination of tendinopathy [33]. Various studies have demonstrated that SWE is a very sensitive and specific method for the detection of changes in tendon structures, and monitoring treatment responses and outcomes in patients with tendinopathies or other tendon pathologies [32,33,34,35,36]. Compared to axial strain elasticity, SWE, which does not require manual compression, is less dependent on operator skills, and therefore allows the more reproducible measurement and evaluation of tendons’ elasticity [37]. 

Symptomatic and painful tendon disorders are a relevant health issue in both the sedentary population and in competitive and recreational athletes. As it is known that there are distinct areas of pathology in disordered tendons, it is even more relevant that SWE can provide direct measurements of specific areas within the tendon. In the clinical setting, shear wave elastography has great potential for use in the early identification of patients at risk of tendon pathology by the use of an easy and fast non-invasive technique [1]. Ultrasound is a valuable diagnostic method to detect tendon abnormalities, which can be proven by biopsy findings [30,38]. Shear wave elastography shows the potential to detect early lateral imbalances [29,39]. Regular pre-season shear wave elastography assessment, along with musculoskeletal check-ups, might help to detect early stiffness changes in sport-specific exposed muscles and tendons. This can result in better individual training control to avoid chronic muscle injuries or tendinopathies. The aim of this study was to investigate the impacts of physical parameters and different sports on patellar tendon stiffness in professional athletes using shear wave elastography.

### 4.1. Influence of Physical Parameters on Patellar Tendon Stiffness

In earlier studies, athletes with patellar tendinopathy were significant younger, but with similar BMIs compared to control groups [1,40]. Overweight and obese individuals have lower patellar tendon stiffness than normal-weight individuals. The body fat percentage seems to be more relevant for PT stiffness than BMI, which has been attributed to metabolic factors, and proinflammatory ones when a higher proportion of adipose tissue is present [41]. It can be assumed that a higher amount of adipose tissue in overweight and obese individuals has a more pronounced effect on patellar tendon stiffness than the increased mechanical load due to body mass [4]. Our results show no influence of BMI in professional athletes or significant differences in BMI between male and female athletes or different sports.

While earlier studies found an age-related decrease in the elastic modulus and SWV of the PT [28], our results do not show a significant impact of age on patellar stiffness in professional athletes. However, our study subjects are younger (18–24.5 years) and are comparable in terms of weekly training hours. Tendons and muscles undergo changes in composition and architecture with aging, which impacts their mechanical properties and function [8,42]. The muscle mass declines with age, leading to a progressive reduction in muscle function and strength. These changes impair the daily life of the elderly [5,43]. However, it has been shown that tendons maintain their dimensions and mechanical properties with aging. Couppé et al. found a lower collagen concentration in the patellar tendon in older men than in younger men, but in turn an age-related increase in enzymatic and nonenzymatic collagen crosslinks [43]. They discussed lower tendon loading due to age-related reduced activity and/or loss of muscle mass and strength as a possible reason for lower collagen concentration, but also physiological age-related tendon changes [43]. Hsiao et al. [37] have shown that shear wave velocity (SWV) can reflect a change in elasticity with age. They report a decrease in the elastic modulus and SWV of the PT in correlation with aging, as well as greater side-to-side differences in older compared with younger participants [37].

Hsiao et al. proposed that SWE might be more useful in identifying signs of tendon abnormalities in comparison to B-mode ultrasonography [37]. Also, young athletes in jumping sports may have a higher risk of patellar tendinopathy and Osgood–Schlatter disease, as patellar tendon stiffness is lower in younger individuals [28]. In young athletes, it is especially important to detect early changes in PT stiffness to prevent the development of Osgood–Schlatter disease and lateral imbalances, as this might lead to long phases without training or consecutive injuries, or an end of a career in professional sports [39,44,45]. Further research should include adolescent athletes to investigate early stages of Osgood–Schlatter disease and patellar tendon stiffness with SWE in order to further explore the potential use of SWE as a preventive tool in the annual basic examination of athletes [46].

The published data on the influence of gender on PT stiffness are inconsistent [4,28]. It is known that a higher mechanical load and training load promote collagen synthesis, which is measurable using SWE [47]. This may explain the higher patellar tendon stiffness in male athletes because of a higher mechanical load due to higher body mass and muscle strength; however, no professional athletes were included in these studies [4,48]. Another explanation could be the effect of female hormones on collagen synthesis. A higher estrogen level leads to a lower estradiol level and reduced collagen synthesis [49]. Higher amounts of adipose tissue have been reported to alter patellar tendon stiffness [48]. Females have a higher body fat percentage, which could be another reason for the lower patellar tendon stiffness in females compared to males, as described by Tas et al. [4]. However, our findings do not show a significant difference in patellar tendon stiffness between female and male professional athletes, suggesting that gender-related differences in patellar tendon stiffness might be reduced by higher mechanical loads in professional sportive activity (e.g., more than 10 training hours per week). It should also be considered that, unlike other tendons such as the Achilles tendon, which are muscle–bone connections, the patellar tendon connects two bone structures. On the other hand, consistent training highly correlates with AT stiffness, for PT load exercise and strength training has not shown an effect of stiffness properties in athletic individuals [40,47,50,51]. Although it is known that different knee angles and quadriceps contraction during examination can affect patellar tendon stiffness [52], there is a lack of standardized protocols. Therefore, it is important to carefully explain this before the examination, and instruct participants to ensure similar conditions are upheld for measurement, such as a consistent knee angle. We propose the use of a roll placed under the knee, as shown in Figure 1. A standardized protocol is important to ensure the comparability of research findings [53,54].

### 4.2. Influnce of Sport Type on Patellar Tendon Stiffness and Clinical Application

Furthermore, our results in the total study population have not demonstrated a significant difference between different types of sports (handball, volleyball, sprint, hammer throw; *p* > 0.05), which is in line with the findings of Esmeili et al. [55]; however, data for large populations of professional athletes are scarce, and further research is necessary.

SWE should be applied more often in athletes with risk factors for patellar tendinopathy owing to difficulties in surgical or conservative treatment and a full rehabilitation [56,57]. We propose seasonal intra-individual longitudinal examinations for detecting patellar tendon changes. A combination of shear wave elastography, power Doppler and B-mode should be applied in professional athletes. Especially, adolescent athletes should be regularly examined, as shear wave elastography can be helpful in detecting lateral imbalances, which are a risk factor for further injuries [39]. 

### 4.3. Measurement Validity of Patellar Tendon Using SWE

SWE can be easily applied as a non-invasive imaging modality, and shows good intra- and inter-reliability [37,58]. Hsiao and colleagues tested the reliability of SWE in a healthy cohort using different anatomical areas of the tendon, defined as the proximal, distal and middle areas. The reliability was excellent at the middle area of the tendon, while the authors noted fair to good results at both anatomical ends (e.g., proximal and distal) [17]. This may be explained by the tendon insertion in an area adjacent to bony structures, where it is more difficult to apply uniform compression over the entire region of interest [59]. SWE performed using the Virtual Touch quantification technique, as used in this study, was both a reliable and repeatable technique for PT stiffness quantification according to intraobserver and interobserver values, as well as intraday measurements [53]. While it is known that optimal transducer placement and uniform pressure applied by the investigator are mandatory in repeated examinations, patient preparation (position, knee angle) also needs to be comparable, since a higher knee angle is associated with higher shear wave speed [40]. Besides the 2D SWE approach used in this study, point SWE (pSWE) also showed excellent reliability with high intraclass correlation in the PT [20]. However, the disadvantage of pSWE is that the point measurement is undertaken without a representative color-coded map, which affects the accurate replication of measurement for each tendon area. 

As mentioned before, estrogen levels have an influence on musculoskeletal tissue [49] and female athletes are still less represented in research [60]. Therefore, we also assessed our female population for the influence of oral contraceptives on patellar tendon stiffness.

### 4.4. Influence of Oral Contraceptives on Patellar Tendon Stiffness

Patellar tendon stiffness in female professional athletes with oral contraceptive intake was higher in comparison to athletes without oral contraceptive intake (OC: 3.723 m/s versus NOC: 3.017 m/s), but it was not significant (*p* = 0.53). Estrogen fluctuations may influence musculoskeletal tissue and collagen synthesis, and fibroblast proliferation may be reduced in the high-hormone phase of the menstrual cycle [49]. As estrogen fluctuation also has an impact on the vasopressin expression pathway [61,62], resulting alterations in tissue fluid might be a reason for the softer tendons seen in our examination. Women taking oral contraceptives do not have endogenous hormone fluctuations [63] and have been found to have more tendon tissue crosslinks, which also leads to higher tendon stiffness [64], and the cross-sectional patellar tendon area was also larger [12]. Differences between cycle phases could not been detected in both groups [12]. Also, we did not measure a difference in menstrual phases between oral contraceptive users and non-users. Furthermore, they did not measure a difference in patellar tendon stiffness in female handball athletes between oral contraceptive-users and non-users [12], which is in line with the findings of this study. Tendon stiffness was calculated, and not gathered using shear wave elastography. Measurement data for tendon stiffness calculation were obtained by ninety-degree knee flexion, thus patellar tendon stiffness can hardly be compared. In addition, Hansen et al. [12] took tendon biopsies and could not find differences in collagen cross-linking and tendon fibril characteristics. The sample sizes in this study and the examinations [12] are comparable but small, and further research is necessary in female athletes. Also, the effect of estrogen on the body’s fluid balance should be further investigated, which might be considered by assessing musculoskeletal tissue [65]. 

### 4.5. Limitations

The subgroups compared to identify the effects of different types of sports were small. Further studies should examine patellar tendon stiffness in larger subgroups of athletes in different sports. Especially, sports with a jumping locomotion pattern and a high risk for patellar femoral pain syndrome needed to be examined multidimensionally, including SWE to avoid chronic pain syndromes. Also, adolescent athletes were not examined in this population. The application of SWE in the prevention of Morbus Osgood–Schlatter should be examined in further studies to avoid longer training absences.

Further studies that examine patellar tendon and tendinopathy should also examine quadriceps and hamstring muscles, as the lower flexibility of these muscles could contribute to patellar tendon pathologies [66]. Regular pre-season examinations are recommended, and the investigation of jumping abilities, especially in adolescent female athletes, should be performed to detect risk factors for patellar tendinopathy [67].

Furthermore, in the subgroup analysis for patellar tendon stiffness and oral contraceptive intake, the number of individuals in the OC group was small. The focus of this study was the examination of the effect of anthropometric parameters on the patellar tendon, though sex hormone levels were not gathered. These are relevant parameters to evaluate, and the results will be investigated in further studies.

## 5. Conclusions

In professional athletes, there are no significant differences in patellar tendon stiffness according to gender, age, BMI and type of sport (handball, volleyball, soccer, sprint, hammer throw). Oral contraceptives may not have an impact on patellar tendon stiffness in female athletes. SWE is a reliable modality to detect abnormal tendon stiffness. Regular longitudinal examinations of the patellar tendon in professional athletes might help to detect early stiffness changes, as an early sign of injury. However, a strict examination protocol is necessary to obtain reliable measurements of tendon stiffness. Further research is necessary to elucidate the value of this technique in preventive programs for professional athletes.

## Figures and Tables

**Table 1:** ANOVA for patellar tendon stiffness.

	Coefficient	SD Error	*p* Value
Bias	−23.843	25.845	0.361
Gender	0.730	0.387	0.065
Age	−0.010	0.34	0.765
BMI	0.572	0.540	0.295
Sports	0.178	0.122	0.152

**Table 2:** Subgroup analysis for gender and PT stiffness.

	Male (*n* = 30)		Female (*n* = 30)	
	Mean and Range		Mean and Range	
PT	3.200	2.844–3.758	3.000	2.536–3.899
Age	18.00	18.00–21.00	20.00	17.75–25.00
BMI	23.38	22.35–24.34	22.49	21.57–25.02

**Table 3:** Subgroup analysis of female athletes with (OC) and without (NOC) oral contraceptive intake.

	Female Athl. NOC (*n* = 21) Mean ± SD	Female Athl. OC (*n* = 9) Mean ± SD	*p* Value
PT	3.017 ± 0.793	3.723 ± 1.061	0.053
Age	21.19 ± 3.80	20.11 ± 4.08	0.509
BMI	22.98 ± 2.96	24.98 ± 3.10	0.122

## Data Availability

The data presented in this study are available on request from the corresponding author. The data are not publicly available due to data privacy regulations.

## Abbreviations

NOC	non-oral contraceptive group
OC	oral contraceptive group
PT	patellar tendon
PFPS	patellofemoral pain syndrome
SWE	shear wave elastography
SWS	shear wave speed
SWV	shear wave velocity
US	ultrasound
